# Risk factors for hospitalizations among patients with cirrhosis: A prospective cohort study

**DOI:** 10.1371/journal.pone.0187176

**Published:** 2017-11-17

**Authors:** Shari S. Rogal, Viyan Udawatta, Imo Akpan, Akshata Moghe, Alexis Chidi, Amit Shetty, Eva Szigethy, Klaus Bielefeldt, Andrea DiMartini

**Affiliations:** 1 Department of Surgery, University of Pittsburgh, Pittsburgh, PA, United States of America; 2 Center for Health Equity Research and Promotion, VA Pittsburgh Healthcare System, Pittsburgh, PA, United States of America; 3 Division of Gastroenterology, Hepatology, and Nutrition, University of Pittsburgh, Pittsburgh, PA, United States of America; 4 Division of General Internal Medicine, University of Pittsburgh, Pittsburgh, PA, United States of America; 5 University of Pittsburgh, School of Medicine, Pittsburgh, PA, United States of America; 6 Department of Psychiatry, University of Pittsburgh, Pittsburgh, PA, United States of America; Chang Gung Memorial Hospital Kaohsiung Branch, TAIWAN

## Abstract

This study was designed to assess unique baseline factors associated with subsequent hospitalizations in a cohort of outpatients with cirrhosis. A cohort of 193 patients with cirrhosis was recruited from an outpatient liver disease clinic at a single, tertiary medical center. Comorbidities, prescription medications, liver disease symptoms and severity, and psychiatric and pain symptoms were assessed at baseline using validated instruments. Inflammatory markers were measured using standardized Luminex assays. Subsequent hospitalizations and the primary admission diagnoses were collected via chart review. Multivariable models were used to evaluate which baseline factors were associated with time to hospitalization and number of hospitalizations. The cohort consisted of 193 outpatients, with an average age of 58±9 and model for end-stage liver disease (MELD) score of 12±5. Over follow-up, 57 (30%) were admitted to the hospital. The factors associated with time to hospitalization included the severity of liver disease (HR/MELD point:1.10, 95% CI:1.04,1.16), ascites (HR: 1.90, 95% CI: 1.01, 3.58), baseline symptoms of depression (HR:2.34, 95% CI:1.28,4.25), sleep medications (HR:1.81, 95% CI:1.01, 3.22) and IL-6 (HR:1.43, 95% CI: 1.10, 1.84). Similarly the number admissions was significantly associated with MELD (IIR: 1.08, CI: 1.07,1.09), ascites (IIR: 4.15, CI:3.89, 4.43), depressive symptoms (IIR:1.54, CI:1.44,1.64), IL-6 (IIR:1.26, CI:1.23,1.30), sleep medications (IIR:2.74, CI:2.57, 2.93), and widespread pain (IIR: 1.61, CI: 1.50, 1.73). In conclusion, consistent with prior studies, MELD and ascites were associated with subsequent hospitalization. However, this study also identified other factors associated with hospitalization including inflammation, depressive symptoms, sleep medication use, and pain.

## Introduction

Hospitalization and readmission rates are rapidly becoming quality markers for patients with chronic illness.[1, 2] Patients with cirrhosis are known to have high rates of healthcare utilization and readmission.[3–5] However, the factors that determine who will be hospitalized with cirrhosis have not been fully elucidated. To date the studies of hospitalization among patients with cirrhosis have focused on medical factors and not on patient symptoms or inflammation. The factors that have been associated with readmission in prior studies have included disease severity (Model for End Stage Liver Disease score, or MELD), demographic factors (age, race), and comorbidities (e.g.; diabetes).[6] For example, one study of 402 patients admitted to the hospital for complications of cirrhosis found that 69% of those patients had subsequent readmission and that readmission was significantly related to MELD, serum sodium, and the number of discharge medications.[5] However, little work has focused on the importance of symptoms and symptom management or on inflammation for hospitalization among patients with cirrhosis.

Inflammation, psychiatric symptoms, and sleep problems are commonly seen in patients with cirrhosis and often cluster together.[7] In a cohort of 1286 consecutive outpatient visitors to our outpatient clinic with varying stages of liver disease we found that the factors significantly associated with 6-month hospitalizations in multivariable modeling included severity of liver disease, cardiopulmonary disease, and chronic opioid usage.[8] However, this was a retrospective, chart review study and was thus unable to assess pain or psychiatric symptoms using validated instruments. The aim of this study was to prospectively assess whether inflammatory markers, pain, and psychiatric symptoms were associated with subsequent hospitalization in a cohort of outpatients with cirrhosis.

## Methods

### Subjects

The University of Pittsburgh’s institutional review board (IRB) approved this study (PRO12060413). A cohort of 210 patients with cirrhosis due to alcohol, hepatitis C virus (HCV), and non-alcoholic steatohepatitis (NASH) was recruited and consented from the outpatient hepatology clinic at the University of Pittsburgh.[9] Patients who had a history of liver transplant, chronic inflammatory conditions (e.g.; multiple sclerosis, malignancies including hepatocellular carcinoma, rheumatologic disorders), or other etiologies of liver disease were excluded, as were patients who could not provide informed consent for any reason (e.g., hepatic encephalopathy, dementia). The diagnosis of cirrhosis was based on clinician diagnosis in the chart and confirmed with review of biopsy results, imaging, and laboratory assessment by the investigators. The determination of encephalopathy was made by the attending hepatologist at the time of recruitment and patients with encephalopathy at that time were excluded to both ensure ability to consent and accuracy of self-reported symptoms. Patients with inflammatory conditions such as cancer were excluded in order to ensure that the inflammatory markers were not being driven by other underlying inflammatory processes. The determination of cirrhosis and the etiology of liver disease were based on reports by the primary treating hepatologist and confirmed with the biopsy, imaging, and laboratory data in the charts. History of prior decompensation was also assessed by chart review. Written informed consent was obtained from participants, and participants completed surveys and serological testing at the index visit.

### Data collection

Surveys included the Hospital Anxiety and Depression Scale (HADS), with a score >8 defining “depression” or “anxiety” on the subscales.[10, 11] Continuous scores were also used in sensitivity analyses. The Pittsburgh Sleep Quality Index (PSQI) was assessed, with a score >5 defining “sleep disorder”[12–14], the McGill Pain Questionnaire (MPQ)[15–17], and the modified American College of Rheumatology 2010 Criteria for Fibromyalgia (ACRF).[18, 19] “Widespread pain” was defined as meeting criteria for fibromyalgia for the purposes of this study. Demographic information was self-reported. Model for End-Stage Liver Disease (MELD) scores were calculated from the laboratory data available closest to the index visit and entered into models as a continuous variable. Medications were obtained from the charts and confirmed with patient reports. Assessment of ascites was based on the clinical assessment in the chart from the index visit. The Charlson Comorbidity Index was calculated for all patients based on comorbidities ascertained using chart review at the index visit.[20, 21]

Blood was assayed using standard Luminex assays at the University of Pittsburgh Cancer Institute Central Laboratory. The samples were run against standardized samples and normalized per established protocols.[22] We evaluated interleukin (IL) 6 and C-reactive protein (CRP).

The charts of the patients were reviewed for 1 year following recruitment in order to ascertain dates and durations of hospitalizations. The primary and secondary discharge diagnoses were recorded.

### Data analysis

All analyses were completed using the R statistical software package, version 2.15.2.[23] The baseline characteristics of the cohort were compared between those participants who were and were not hospitalized during follow up using Student’s t-tests and chi-square tests for continuous and categorical variables respectively. Because the distributions of the inflammatory markers were non-normal, testing was completed using Wilcoxon Rank-Sum testing. Those covariates with a p<0.2 in univariate testing were included in the full logistic and competing risk models of any hospitalization time to the first hospitalization. The competing risk model included death as a competing risk and was reduced using backwards elimination. The logistic regression and hospitalization models were reduced using that MASS package [24], which optimized Akaike’s Information Criterion in order to create a parsimonious final model. The number of hospitalizations was evaluated with negative binomial regression using follow-up time as an offset. Secondary models were assessed in order to evaluate whether the number of admissions in the year prior to the study impacted the models. A high admission group was *a priori* defined as being in the top quartile of number of admissions in the admitted group, which was ≥5 in this cohort. This group was assessed for differences in baseline characteristics vs. the lower admission group vs. the no admission group. Chi-square, Fisher’s exact, ANOVA, and Kruskal-Wallis tests were used to compare the differences in the baseline characteristics between these 3 groups. IL-6 and CRP were initially used in modelling together and then CRP was entered into models alone.

## Results

The final cohort consisted of 193 outpatients, with an average age of 58±9 and MELD of 12±5 (S1 Dataset). The most common etiology of cirrhosis in this cohort was HCV (n = 78, 40%) followed by NASH (n = 66, 34%) and alcohol (n = 49, 25%). Of these 193 patients, 57 (30%) were admitted to the hospital over the follow-up period. The number of admissions over follow-up ranged from 0–14. There were 8 deaths over follow-up, including 2 in-hospital deaths during index admissions.

### Reasons for admissions

Patients with cirrhosis were admitted when they experienced complications of their liver disease or other conditions that could not be managed in the outpatient setting. Of the 54 initial admissions with available discharge diagnoses for review (95%), 21 were liver-related admissions (39%). When assessing primary discharge diagnoses, 16 were infection-related (6 for cellulitis, 4 for pneumonia, 4 for bacteremia, 1 for osteomyelitis, and 1 for gastroenteritis) and 13 admissions were for renal, electrolyte, or fluid complications (6 for ascites, 2 for hepatic hydrothorax, 5 for other), 7 were for encephalopathy, and 8 were for bleeding (3 for variceal bleeding, 5 for other bleeding). Ten of the admissions were for other reasons including 2 for orthopedic issues and one for each of the following reasons: liver transplantation, pulmonary hypertension, abdominal pain, accidental dialysis catheter removal, transfusion reaction, deep vein thrombosis, seizure, and facial weakness.

### Univariate analysis

Table 1 illustrates the baseline characteristics of patients requiring admission compared to those patients not requiring admission. Those patients who had a subsequent hospitalization had significantly higher MELD scores and were more likely to have ascites. This group also had higher rates of baseline depressive symptoms and increased use of sleep medications at baseline. The levels of IL-6 and CRP were also significantly higher in this group at baseline. The groups with and without admission notably had the same baseline Charlson comorbidity index scores.

**Table 1:** Baseline characteristics by hospitalization status.

	Hospitalization N = 57	No Hospitalization N = 136	P
*Demographics*			
**Age** (mean±sd)	58±8	58±9	0.83
**Female** (n, %)	20 (35)	57 (42)	0.47
**Non-white race** (n, %)	5 (9)	9 (7)	0.84
*Liver Disease*			
**MELD** (mean±sd)	15±6	11±4	<0.01
**Ascites** (n, %)	41 (72)	52 (38)	<0.01
**Etiology** (n, %)			0.58
**HCV**	23 (40)	55 (40)	
**ETOH**	17 (30)	32 (24)	
**NASH**	17 (30)	49 (36)	
*Comorbidities*			
**Depression** (n, %)	31 (54)	38 (28)	<0.01
**Widespread Pain** (n, %)	20 (35)	32 (24)	0.14
**Sleep Disorder** (n, %)	54 (95)	121 (89)	0.28
**Comorbidity Index** (mean±sd)	4±1	4±1	0.99
**Diabetes** (n, %)	19 (33)	44 (32)	1.00
**Prescription Opioids** (n, %)	15 (26)	30 (22)	0.65
**Sleep Medications** (n, %)	26 (46)	37 (27)	<0.01
*Inflammatory Markers*			
**IL-6** (pg/mL, median iqr)	4.63 (1.52,7.20)	1.60 (0.92,4.44)	<0.01
**CRP** (mg/dl, median,iqr)	1.8 (0.8,2.9)	1.2 (0.5,2.9)	0.03

## Multivariable analysis

The factors at baseline that were significantly associated with hospitalization in the multivariable logistic regression model are shown in Table 2 and included MELD, ascites, depressive symptoms, taking baseline sleep medications, and IL-6 levels. All factors remained significant in the multivariate competing risk model, excluding IL-6. The factors significantly associated with the number of hospitalizations over follow-up are shown in Table 3 and included liver disease severity in the form of MELD and ascites as well as depression, sleep symptoms, widespread pain, IL-6, and the use of sleep medications. There was no evidence of collinearity found in any model. Of the 193 patients included in the study, 62 (32%) had been hospitalized in the year prior to enrollment. The number of admissions in the year prior to the study was associated with subsequent admissions in all models; including this variable did not change the significance of the variables in the final models. Entering the HADS depression score as a continuous variable rather than using a threshold of 8 did not change the models. When CRP and IL-6 were used in models together, CRP was not significantly associated with outcomes. However, when IL-6 was removed from the models and CRP alone was substituted, the models did not substantially change and CRP *was* significantly associated with number of hospitalizations (IRR = 1.11, 95% CI = 1.08,1.13) but not hospitalization or time to hospitalization.

**Table 2:** Logistic regression and Cox proportional-hazard regression models of hospitalizations.

	Time to Hospitalization			Any Hospitalization		
Covariate	HR	95% CI	P	OR	95% CI	P
**MELD**	1.10	1.04,1.16	<0.01	1.12	1.03,1.22	0.01
**Ascites**	1.90	1.01,3.58	0.047	2.19	0.99,4.89	0.05
**Depression (HADS>8)**	2.34	1.28,4.25	<0.01	2.58	1.21.5.54	0.01
**Sleep medications**	1.81	1.01,3.22	0.046	2.63	1.19,5.97	0.02
**IL-6**	1.43	1.10,1.84	<0.01	1.41	0.95,2.19	0.10

**Table 3:** Negative binomial regression models of number of hospital admissions.

	Number of Hospitalizations	
Covariate	IRR	95% CI
**MELD**	1.08	1.07,1.09
**Ascites**	4.15	3.89,4.43
**Depression (HADS >8)**	1.54	1.44,1.64
**Sleep Disorder (PSQI>5)**	1.08	0.97, 1.21
**Widespread Pain**	1.61	1.50, 1.73
**Sleep medications**	2.74	2.57,2.93
**IL-6**	1.26	1.23,1.30

### High risk admission group

A small percentage of the patients (n = 14, 7%) accounted for a large percentage (56%) of admissions over follow-up. As shown in Table 4, this group had significantly higher MELD scores, CRP and IL-6 as well as an increased likelihood of ascites, depressive symptoms, sleep medication use, and prescription opioid use vs. the lower admission and no admission groups.

**Table 4:** High admission group vs. other groups of patients.

	Over 4 Hospitalizations	≤4 Hospitalizations	No Hospitalizations	P
	N = 14	N = 43	N = 136	
*Demographics*				
**Age** (mean±sd)	58±8	60±8	58±9	0.19
**Female** (n, %)	6 (43)	14 (33)	57 (42)	0.54
**Non-white race** (n, %)	2 (14)	3 (7)	9 (7)	0.12
*Liver Disease*				
**MELD** (mean±sd)	19±6	13±5	11±4	**<0.01**
**Ascites** (n, %)	13 (93)	28 (65)	52 (38)	**<0.01**
**Etiology** (n, %)				0.78
**HCV**	6 (43)	17 (40)	55 (40)	
**ETOH**	5 (36)	12 (28)	32 (24)	
**NASH**	3 (21)	14 (33)	49 (36)	
*Comorbidities*				
**Depression** (n, %)	6 (42)	25 (58)	38 (28)	**<0.01**
**Widespread Pain** (n, %)	6 (42)	14 (33)	32 (24)	0.14
**Sleep Disorder** (n, %)	14 (100)	40 (93)	121 (89)	0.49
**Comorbidity Score** (mean±sd)	4±1	3±1	4±1	0.31
**Diabetes** (n, %)	2 (14)	17 (40)	44 (32)	0.22
**Prescription Opioids** (n, %)	7 (50)	8 (19)	30 (22)	**0.04**
**Sleep Medications** (n, %)	7 (50)	19 (44)	37 (27)	**0.04**
*Inflammatory Markers*				
**IL-6** (pg/mL, median iqr)	9.87 (3.00,33.65)	4.00 (1.41,5.90)	1.60 (0.92,4.44)	**<0.01**
**CRP** (mg/dl, median,iqr)	2.5 (1.6,5.0)	1.5 (0.8,2.7)	1.2 (0.5,2.9)	**0.04**

## Discussion

Using a large cohort of patients with known cirrhosis receiving outpatient care in a tertiary referral center, we examined potentially modifiable predictors of future need for hospitalization. Consistent with prior reports, the severity of liver disease, a well-established risk factor for hospitalization in this population, significantly increased the risk of admission for liver-related or other medical problems. [4–6] However, several other novel factors independently correlated with hospitalizations, including elevated inflammatory markers at the time of the study enrollment, depressive symptoms, pain, and sleep medication use. These associations may help to identify a high-risk groupfor early intervention, potentially allowing us to prevent hospitalization. Prior studies consistently showed that a small minority of patients consume a disproportionate amount of healthcare resources, a pattern that has also been described in cirrhosis and in this study, where 7% of patients accounted for 53% of admissions. This group with high resource utilization had more advanced liver disease in terms of MELD score and ascites, which was expected. However, this study also found that this group was more likely to take prescription opioid and sleep medications. Those patients accounting for the majority of admissions also had increased depressive symptoms and much higher IL-6 and CRP levels. While the group size did not allow a meaningful multivariate analysis these each have biological plausibility and serve as potential markers for patients in need of early interventions. [25]

We also examined serologic markers of inflammation as potential biomarkers for subsequent hospitalization risk. These results confirm the potential utility of CRP in this context. CRP has been previously evaluated as a prognostic marker for short-term mortality in patients with cirrhosis.[26] CRP levels were found to improve MELD score accuracy. [26–28] Given that CRP is widely available and inexpensive, this may be considered for routine integration into clinical care. A second potential biomarker, IL-6, has been associated with the development of hepatic fibrosis. [29, 30] Moreover, the levels of pro-inflammatory cytokines have been reported to be elevated among patients with cirrhosis compared to healthy controls, with elevations of IL-6 found in both compensated and decompensated cirrhosis.[29] Our group and others have previously reported an association between IL-6 and/or CRP and anxiety, depression, poor sleep quality and pain.[9, 31, 32] Our findings thus extend these results by suggesting a potential utility of inflammatory markers in predicting hospitalization. Future work should determine how inflammatory biomarkers can be integrated into the care of patients with cirrhosis.

We found a significant association between depressive symptoms and hospitalization in this cohort of patients with cirrhosis. Depression has previously been associated with admissions and length of stay in other disease processes. [33–35] This association is likely multifactorial. Depression is associated with cardiovascular disease and stroke and may thus increase the overall disease burden.[36] Depression has also been associated with somatization [37] which can lead to increased healthcare seeking behaviors[38] with medication non-adherence [39], and inflammation.[40] Given these associations, depression screening and intervention in hepatology clinics may aid in the overall care and hospital prevention for patients with cirrhosis.

Sleep disturbances are common among patients with liver disease, related to both the associated inflammation [41] and encephalopathy[42]. Independent of hepatic encephalopathy, patients with cirrhosis have dysregulated levels of melatonin[42, 43] which can result in excessive fatigue.[44] Conversely, while hypnotics and sedatives can help promote sleep, they can precipitate hepatic encephalopathy.[41] This is particularly true of benzodiazepines.[45] and over-the-counter agents, such as anti-histamines.[46] Nearly half of hospitalized patients in our study were taking sleep medications compared to less than one third of non-hospitalized patients. It is notable that the majority of the patients in the study met criteria for sleep disorders and none were considered to be encephalopathic at the time of study enrollment. Future work should include an assessment of how sleep disorders, as measured by the PSQI, map onto minimal hepatic encephalopathy and whether aggressive treatment of minimal hepatic encephalopathy reduces symptoms of sleep disturbance or the need to use sleep medications. Overall these results also suggest a role for evidence-based behavioral therapies for sleep-related symptoms rather than the use of pharmacotherapies in this population.

Consistent with our prior retrospective study [8], widespread pain was associated with an increased number of hospitalizations in this prospective assessment. Comparable results have been reported in patients with inflammatory bowel disease and other acute and chronic conditions.[33, 47] Providers often have difficulty addressing pain in this population with contraindications to several classes of traditional analgesics. Considering the impact of pain on quality of life and–as highlighted with this study–resource utilization, there is an urgent need to develop evidence-based pain management strategies for patients with liver disease that effectively alleviate suffering while minimizing risks of adverse events.

The complexity and interrelationship of predictors, ranging from inflammation, pain, and sleep disorders to depressive symptoms in this population with cirrhosis points at the potential value of an integrated, team-based approach to the care of patients with cirrhosis. This approach has been shown to decrease healthcare utilization in both primary care and hepatology practices.[25, 48] Simple and effective screening tools to to identify patients in need of increased attention and interventions could be developed if these results are validated in larger cohorts. Routine, critical review of prescription, non-prescription and over-the-counter use of pharmaceuticals may help prevent hospitalizations in the setting of cirrhosis. Behavioral approaches to improve sleep hygiene and proactive interventions through care managers should focus on patient subpoulations identified by this and other studies. Future studies are needed to determine if implementation of such strategies indeed improve health and decreases resource utilization.

While these prospective findings contribute to a growing understanding of the factors associated with hospitalization and the role of inflammation in cirrhosis, there are several limitations of this study. This single-center study will require validation in a larger, multi-center cohort in order to create more generalizable predictive models. Despite the relatively large patient group, sample size limitations precluded an assessment of less common comorbidities and other etiologies of liver disease. Additional limitations include that the measures were assessed at one time point and not serially or at the time of hospitalization and that clinician report of cognitive symptoms and encephalopathy was used rather than a standardized instrument. This study focused on the most common etiologies of cirrhosis and may not apply to patients with other etiologies. Medical intensive care unit (MICU) admission criteria include unstable vital signs, hemodynamically significant bleeding, or other medical conditions that cannot routinely be managed in the medical ward. Though the study was not designed to assess MICU admissions, patients with variceal bleeding and sepsis that were included in the study were admitted to the MICU. Future studies with larger numbers of admitted patients may include an assessment of the factors associated with MICU admissions. Despite these limitations, this prospective study, with validated instruments coupled with inflammatory measures provides new insights into hospitalizations among patients with cirrhosis.

In summary, the present study suggests that several factors are associated with increased hospitalizations in cirrhotic patients. In addition to measures of disease severity, symptoms of depression, inflammation, and the use of sleep medications were associated with subsequent hospitalizations in outpatients with cirrhosis. Future studies should assess whether interventions aimed to reduce these risk factors can help reduce the high number of admissions for patients with cirrhosis.

## Supporting information

S1 Dataset (CSV)
